# Prediction of pyramidal tract side effect threshold by intra-operative electromyography in subthalamic nucleus deep brain stimulation for patients with Parkinson's disease under general anaesthesia

**DOI:** 10.3389/fsurg.2024.1465840

**Published:** 2024-10-10

**Authors:** Lok Wa Laura Leung, Ka Yee Claire Lau, Kwok Yee Patricia Kan, Yikjin Amelia Ng, Man Chung Matthew Chan, Chi Ping Stephanie Ng, Wing Lok Cheung, Ka Ho Victor Hui, Yuen Chung David Chan, Xian Lun Zhu, Tat Ming Danny Chan, Wai Sang Poon

**Affiliations:** ^1^ Department of Neurosurgery, Prince of Wales Hospital, Chinese University of Hong Kong, Hong Kong, Hong Kong SAR, China; ^2^ Department of Anaesthesia and Intensive Care, Prince of Wales Hospital, Chinese University of Hong Kong, Hong Kong, Hong Kong SAR, China

**Keywords:** electromyography, anesthesia, pyramidal tract, Parkinson’s disease, subthalamic nucleus, deep brain stimulation

## Abstract

**Introduction:**

In DBS for patients with PD, STN is the most common DBS target with the sweet point located dorsal ipsilaterally adjacent to the pyramidal tract. During awake DBS lead implantation, macrostimulation is performed to test the clinical effects and side effects especially the pyramidal tract side effect (PTSE) threshold. A too low PTSE threshold will compromise the therapeutic stimulation window. When DBS lead implantation is performed under general anaesthesia (GA), there is a lack of real time feedback regarding the PTSE. In this study, we evaluated the macrostimulation-induced PTSE by electromyography (EMG) during DBS surgery under GA. Our aim is to investigate the prediction of post-operative programming PTSE threshold using EMG-based PTSE threshold, and its potential application to guide intra-operative lead implantation.

**Methods:**

44 patients with advanced PD received STN DBS under GA were studied. Intra-operative macrostimulation via EMG was assessed from the contralateral upper limb. EMG signal activation was defined as the amplitude doubling or greater than the base line. In the first programming session at one month post-operation, the PTSE threshold was documented. All patients were followed up for one year to assess clinical outcome.

**Results:**

All 44 cases (88 sides) demonstrated activations of limb EMG via increasing amplitude of macrostimulation the contralateral STN under GA. Revision tracts were explored in 7 patients due to a low EMG activation threshold (<= 2.5 mA). The mean intraoperative EMG-based PTSE threshold was 4.3 mA (SD 1.2 mA, Range 2.0–8.0 mA), programming PTSE threshold was 3.7 mA (SD 0.8 mA, Range 2.0–6.5 mA). Linear regression showed that EMG-based PTSE threshold was a statistically significant predictor variable for the programming PTSE threshold (*p* value <0.001). At one year, the mean improvement of UPDRS Part III score at medication-off/DBS-on was 54.0% (SD 12.7%) and the levodopa equivalent dose (LED) reduction was 59.5% (SD 23.5%).

**Conclusion:**

During STN DBS lead implantation under GA, PTSE threshold can be tested by EMG through macrostimulation. It can provide real-time information on the laterality of the trajectory and serves as reference to guide intra-operative DBS lead placement.

## Introduction

Subthalamic Nucleus (STN) is the most common target of Deep Brain Stimulation (DBS) for patients with Parkinson's Disease (PD). STN is a small nucleus located just medial to the pyramidal tract, the main motor pathway controlling voluntary motor activity. The sweet point of STN stimulation has been reported in literature to be located in the dorsolateral part of the nucleus (1). This poses implications for DBS lead implantation, as an overly lateral positioned DBS electrode may generate a pyramidal tract side effect (PTSEs) at a lower stimulation threshold, compromising the efficacy of therapeutic stimulation. The most common PTSEs are dysarthria, contralateral facial spasm, gaze deviation, and contralateral limb muscle contraction (2). As PTSEs are common and disabling adverse effects in chronic STN stimulation, it is important to identify the electrical threshold at which PTSEs occur during the operation. The threshold should ideally be found at a level higher than the optimal therapeutic window (2). The effective STN stimulation is usually in the range of 2–4 mA, and thus the PTSE threshold should not be lower than 4 mA (3).

In traditional DBS lead implantation, surgery is performed whilst the patient is awake. A stimulation test is performed to test both the clinical and side effects intra-operatively, thereby allowing for precise placement of the DBS electrode with an adequate therapeutic window. However, the surgery itself requires a highly cooperative patient who has the physical and psychological endurance to withstand the conditions of awake surgery. With the advancement of pre-operative imaging modalities, surgical planning soft wares and improvement in lead placement technique, there has been a worldwide trend towards performing DBS under asleep or general anesthesia (GA) conditions (4–9). However, when comparing DBS in awake patients with clinical macrostimulation vs. asleep DBS with or without limited test stimulation, the latter demonstrated high prevalence of motor side effects, suggesting potential chance of suboptimal lead positioning (10). A recent meta-analysis also showed that a greater number of stimulation side effects were found in patients whom underwent DBS surgery in asleep vs. awake procedures (7).

It is important for us to consider both the comfort of patients undergoing DBS surgery vs. the ability for surgeons to maintain the efficacy of DBS surgery. Currently, there are a variety of anaesthesia options which allows for clinical neurophysiological evaluation such as sleep-awake-sleep for MER and macrostimulation (11), observation of muscle contraction during macrostimulation in asleep conditions (12, 13). At present, many studies have looked into methods of PTSE prediction and avoidance through the study of distance between the active contact and the pyramidal tract, the relationship of volume of tissue activated by DBS lead with the pyramidal tract, the cortical evoked potential induced by pyramidal tract activation, tractography patterns and EMG patterns (2, 14–22). Yet, to our best knowledge, there is a lack of literature involving studies which provide direct feedback of the pyramidal side effect during the DBS lead placement under GA (23). Our hypothesis is that PTSE can be detected by EMG during STN DBS operation under GA. The EMG-based PTSE threshold guides the intra-operative lead placement to predict the clinical PTSE threshold in subsequent programming.

## Methods

### Patients

All patients fulfilled the Queen Square Brain Bank Criteria for diagnosis of PD and experienced significant motor complications despite maximal medical therapy. Prior to surgery, each patient was evaluated for suitability for DBS by a specialized Movement Disorder Group comprised of neurologists, neurosurgeons, radiologist, clinical psychologist, a nurse specialist and various allied health members. The assessment included clinical UPDRS with levodopa challenge test, neuroimaging, neurocognitive tests and psychiatric consultation. Patients whom met the below criterion were considered for GA-based DBS procedure, due to the likely intolerance towards a LA-based procedure. Our criteria included, (1) significant anxiety or depression as per assessment of the clinical psychology team and (2) known underlying psychiatric disorders that requires active psychiatric follow up although not considered a contraindication for DBS implantation. In those whom met the above criteria, GA-based DBS procedure was recommended. For those who did not meet the above criterion, both LA-based and GA-based DBS implantation were the options as the patient preferred. The clinical outcome was assessed at one year post-operatively. The study protocol was approved by CUHK-PWH ethics committee (IRB Reference Number: 2022.479). Informed consent was obtained from all patients.

### Imaging acquisition and target planning

Magnetic resonance imaging (MRI) 1.5 T or 3 T of the brain was acquired before the operation, including T1 three-dimension (3D), T2 axial, coronal and sagittal 2 mm fine cut, 3D susceptibility weighted imaging (SWI) and diffusion tractography imaging (slice thickness 2 mm, Acquisition matrix 112 × 112, 32 direction). The software for target and trajectory planning were iPlan or Elements (BrainLab, Feldkirche, Germany). The target was marked at the centre of STN corresponding to the anterior border of red nucleus at T2 axial image. The preliminary X/Y/Z coordinates were 12–13 mm lateral, 2–3 mm posterior and 3–4 mm inferior to the mid-commissural point respectively. The entry point was 2 to 4 mm from midline and at 1–2 cm anterior the coronal suture. Fine adjustment of the trajectory was made to avoid nearby sulcus and vessels. On T2 coronal view, the target was adjusted to 2.5 to 3 mm medial to the medial edge of the pyramidal tract using tractography data as reference. In patients whom received surgery after 2020, targeting was also guided by the auto-segmented STN by the Elements software. On the day of operation, a computed tomography (CT) of the brain (helical scan, 0.625 per slice,) was performed with the stereotactic frame (Leksell G frame, or Leksell Vantage frame since 2000) fixed to the head. The CT was then fused to the MRI plan and the frame coordinates were generated by the planning software.

### Surgical procedure

**Figure 1:**
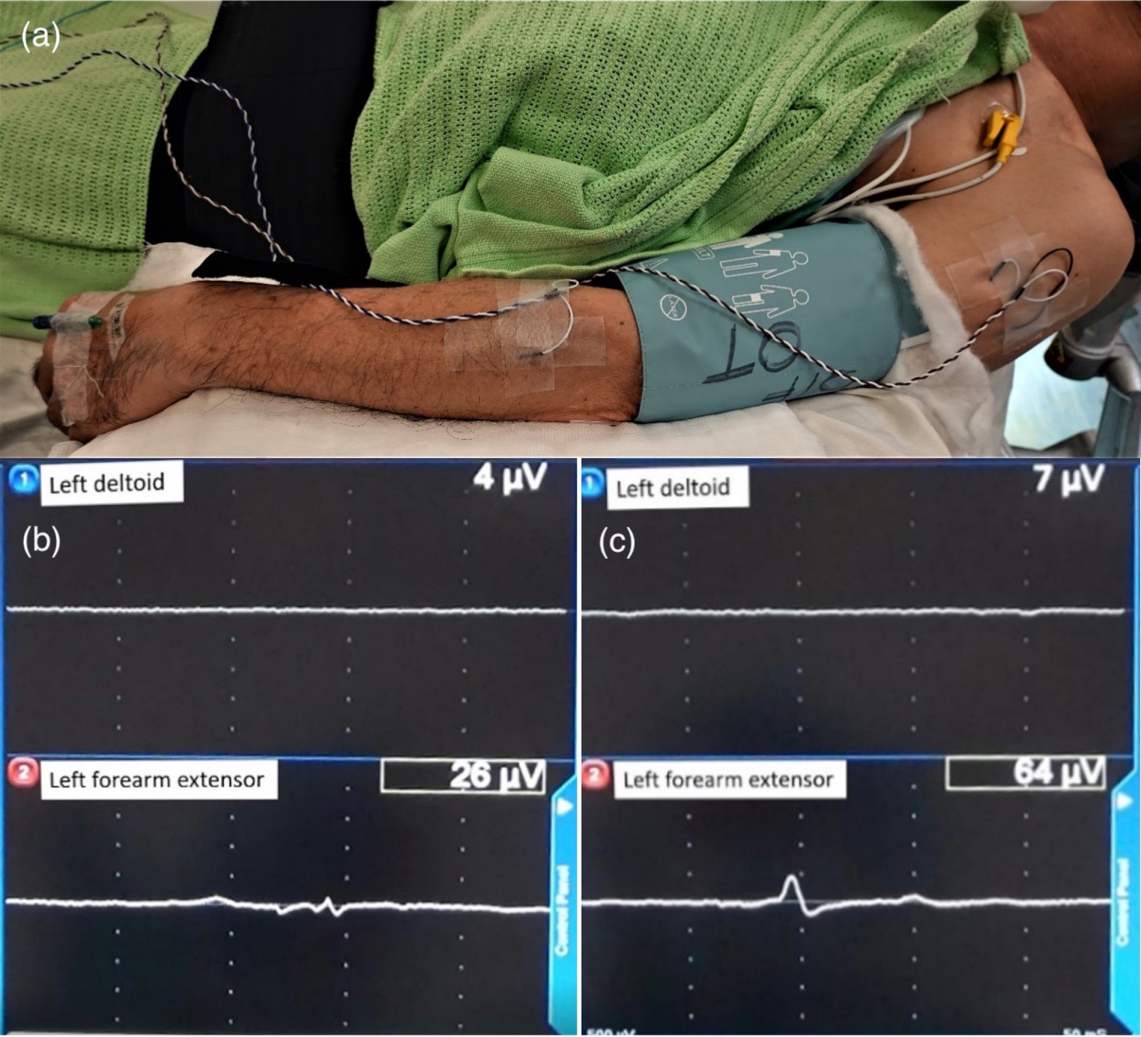
Illustration of EMG patient setup and EMG activation pattern. **(a)** Image illustrating the placement of needles for EMG monitoring into the deltoid and forearm extensor muscle group of the left arm. **(b)** Baseline EMG signal of 26 µV as detected on EMG monitoring. **(c)** Intra-operative EMG-based side effect threshold on EMG monitoring, as detected by equal or double the baseline amplitude of EMG signal.

All patients were off dopaminergic medication for at least 12 h before surgery. Bilateral DBS lead and pulse generator implantation surgery was performed under GA with tracheal intubation. All patients were given total intravenous anesthesia with propofol and remifentanil. Bispectral index (BIS) monitoring was used as guidance for depth of anaesthesia. Propofol concentrations were targeted between 2–6mcg/ml using the pharmacokinetic Marsh model. Remifentanil dose ranged from 0.01–0.5mcg/kg/min intraoperatively. Muscle relaxants was stopped after an initial induction dose to facilitate intubation of the patient and transport of patient for CT. The patient was positioned in supine with the head in neutral position. Needle EMG was set up to the upper limbs of the deltoid and forearm extensor muscle groups (Figure 1a). A burr hole was made and a microelectrode was inserted for microelectrode recording (MER). After MER, monopolar macrostimulation was performed through the macro contact of the electrode. The site of macrostimulation was at the location with best MER signal and the site was documented by its distance from the target along the tract. EMG signal response was detected from the upper limbs muscle groups, including deltoid and forearm extensors contralateral to the side of stimulation (Figures 1b,c). The stimulation began at 0.5 mA/60 uS/130 Hz. The intensity of stimulation was gradually increased by incrementations of 0.5 mA until the EMG signal was activated defined as the amplitude doubling or greater than the base line (Figures 1b,c). This was documented as the intra-operative EMG-based side effect threshold. During each step of stimulation mA increment, we also looked for signs of facial asymmetry, eyes deviation or pupillary change. Bispectral index (BIS) was maintained between 40 and 60 at all times during MER and macrostimulation. Targeted propofol concentration was titrated down, as guided by BIS, to optimize MER/EMG recordings. If the EMG-based side effect threshold was less than 3 mA, alternative tract (s) away from the pyramidal tract were explored. After the appropriate trajectory was selected, the microelectrode was removed and the DBS lead (Medronic 3389 or Medronic directional lead or Vercise Cartesia directional lead, Boston scientific) was implanted. The procedure was performed on the right side first followed by the left side. The pulse generator was implanted during the same operation.

### Post-operative follow-up

At one month after the operation, a CT brain was performed and fused with pre-operative MRI plan. The DBS lead and contacts were marked manually based on the CT image, or recognized by the planning software (BrainLab Element). During the same follow-up session, the first DBS programming was performed by a nurse specialist with the patient at medication Off for 12 h status. Monopolar stimulation of each DBS lead contact was tested individually with 0.5 mA incrementations in frequency 130 Hz and pulse width 60uS. The effect and PTSE threshold were documented. Contralateral motor symptoms were regarded as pyramidal side effects, including bilateral contralateral eye deviation, and/or contralateral facial or limb muscle spasm. The clinical side effect threshold generated during programming session was defined as the generated side effect threshold in mA of a contact closest to the intraoperative macrostimulation site. Finally, the contact with best therapeutic window was chosen as the active contact for chronic stimulation. Patients were then seen at regular intervals for optimization of stimulation parameters and for medication titration guided by the neurologist of the team. Clinical outcomes were assessed at one year post-operatively including UPDRS, QOL (PD39), LED and neuropsychology assessment.

### Statistical analyses

All statistical analyses was performed on the Statistical Package for the Social Sciences [Windows version 26.0; SPSS Inc, Chicago (IL), US]. Paired student *t*-test and linear Regression was used to assess the relationship between the intra-operative EMG threshold vs. post-operative clinical threshold. A *p*-value of 0.05 was used for statistical significance.

## Results

### Demographics

There were 44 consecutive patients with Parkinson’s disease received DBS STN under GA between January 2018 to February 2023 (18 males, 26 females, mean age 60.7, range 42–73 years old) (Table 1).

**Table 1:** Patient demographics and clinical outcomes at one-year post-operation.

Characteristic	Value
Gender	
Male	18 (41%)
Female	26 (59%)
Age at operation	
Mean (SD, Range)	60.7 (7.2, 42–73)
UPDRS part III score	
Pre-Operative Med Off	
Mean (SD, Range)	38.6 (13.9, 13–87)
Pre -Operative Med ON	
Mean (SD, Range)	17.9 (7.2, 6–34)
Pre-Operative Dopamine challenge test improvement	
Mean (SD, Range)	52.7% (12.7, 30.7–75.0)
Post-operative one year Med OFF/DBS OFF	
Mean (SD, Range)	45.8 (14.3, 19–81)
Post-operative one year MED OFF/DBS ON	
Mean (SD, Range)	21.7 (10.4, 5–53)
Post-Operative one-year improvement (Med Off & DBS On):	
Mean (SD, Range)	54.0% (12.7, 27.4–79.6)
Post-operative one-year L-dopa equivalent dosage reduction	
Mean (SD, Range)	59.5% (23.5, 13.4–92.1)

### EMG-based and post-operative programming PTSE threshold

**Figure 2:**
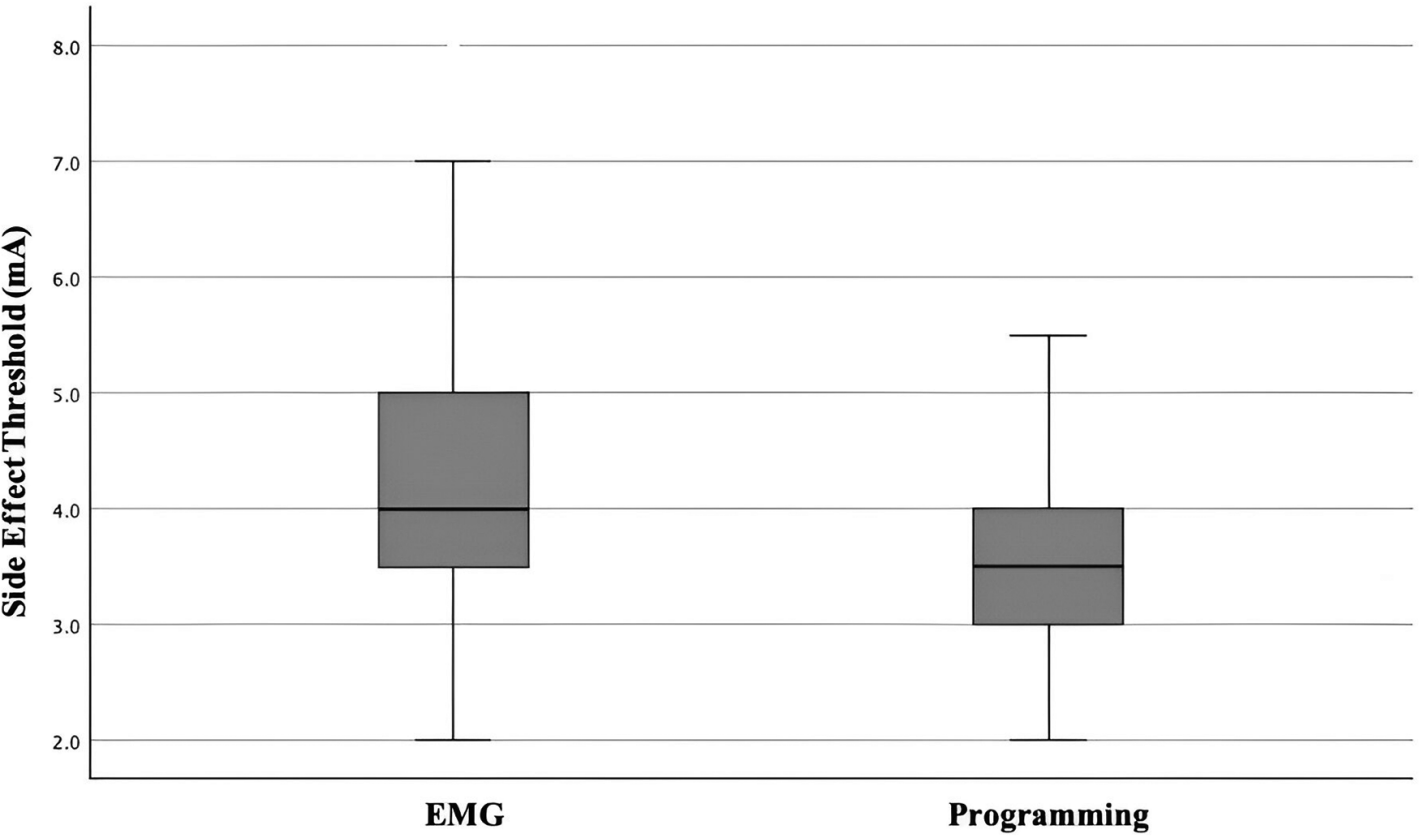
Box and whiskers chart demonstrating EMG-based PTSE threshold vs post-operative programming PTSE threshold.

All 44 cases (88 sides) demonstrated activations of EMG of the upper limbs with progressively increased voltage of contralateral stimulation. As illustrated in Figure 2, the mean voltage of intra-operative EMG-based PTSE threshold was 4.3 mA (SD 1.2 mA, range 2.0–8 mA). The mean voltage of post-operative programming PTSE threshold was 3.7 mA (SD 0.8 mA, range 2.0–6.5 mA), with a mean difference of 0.6 mA (SD 1.1 mA, range −=2.0–4 mA). Post-operatively, the majority of the side effects involved contralateral facial spasm, slurring of speech, or gaze deviation towards the contralateral side. During the operation, 7 image-based tracts were repositioned amongst the 88 tracts (8%) due to a low EMG-based PTSE threshold. The direction of change was mainly medially and/or anteriorly along the oblique axis of the STN on axial view (24).

Among the 88 pairs of EMG-based PTSE vs. post-operative programming PTSE thresholds, the EMG-based PTSE threshold was equal or higher than that obtained by programming in 67 pairs (67%). Within these 67 pairs, 60 (90%) were found within a range of 0–2 mA, and 7 (10%) were found within a range of 2.5–4 mA. In the remaining 21 pairs (24%), the programming PTSE threshold was higher than EMG-based PTSE threshold, all within 1 mA except 2 pairs with 1.5 mA difference and 1 pair with a 2 mA difference.

We noted that all EMG-based PTSE threshold had a value of >3 mA in all 88 pairs with the exception of 3 pairs. The lowest value noted was 2.0 mA, whilst the highest value was found to be 8 mA. Within this specific case whereby the EMG-based PTSE threshold was found to be low, we accepted this intra-operative result in view of the excellent MER signal obtained intra-operatively. Despite the low threshold encountered intra-operatively, we hoped to mitigate such findings with bipolar stimulation post-operatively. During post-operative programming, the PTSE threshold was found to be 3 mA. At the one year follow up, the stimulation parameters were left side 2.8 mA (the side with EMG-based PTSE threshold 2.5 mA), right side 3.3 mA, pulse width 60uS and frequency 100 Hz for both sides. The clinical outcomes of this case at one year were UPDRS part III improvement of 43% with DBS on only, and 74% with both medication and DBS on. The LED reduction rate was 59%.

**Figure 3:**
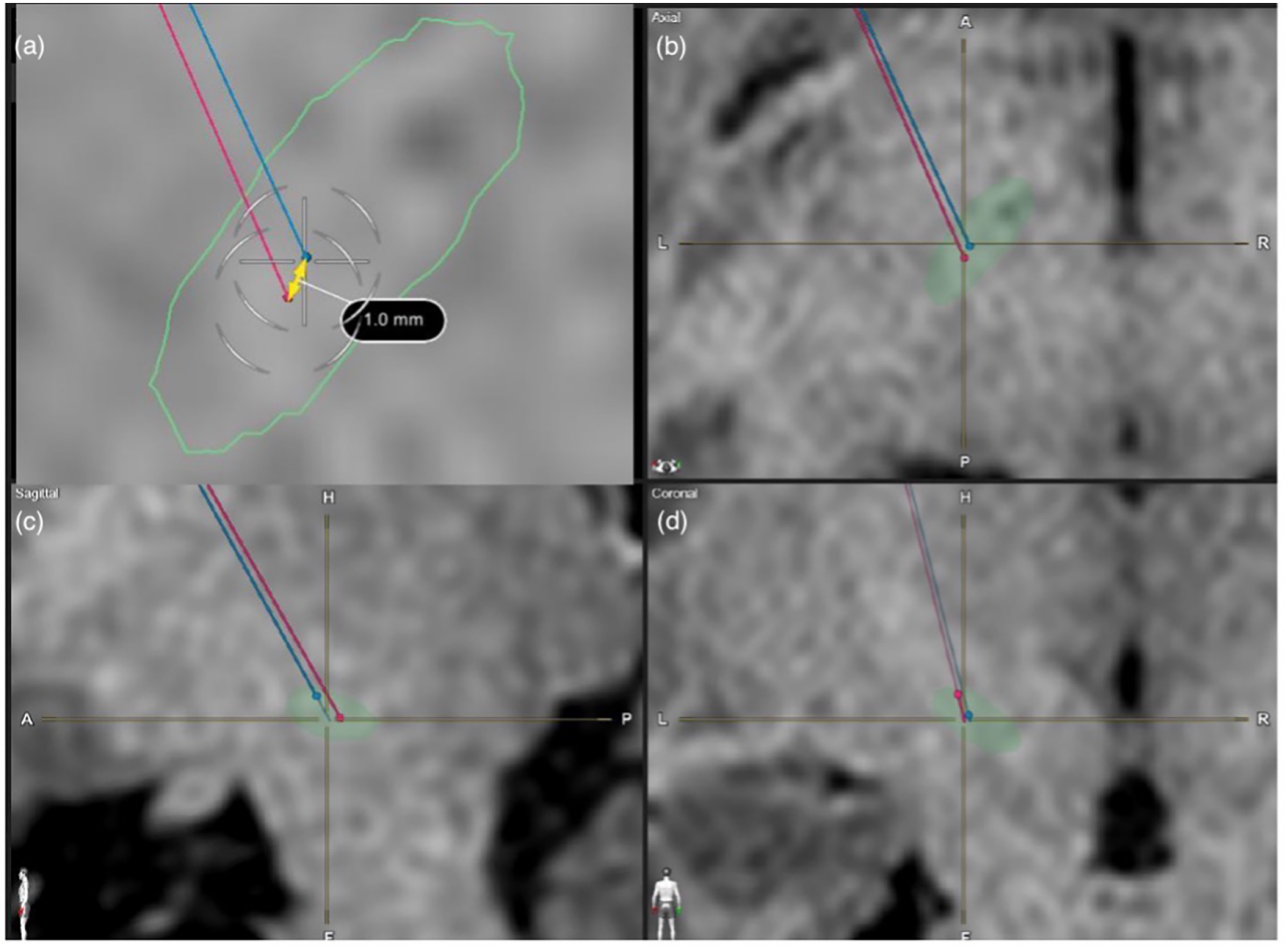
MRI images showcasing a case study whereby the image-based lead trajectory was altered in view of the low EMG-based PTSE threshold. Shown here are T1 sequence MRI Brain images used for lead trajectory planning for STN-based DBS surgery. **(a)** axial view, **(b)** enlarged axial view, **(c)** sagittal view, **(d)** coronal view. The auto-segmented STN by the Brainlab Elements program was outlined in green. The red trajectory was the original planned image-based trajectory. The blue trajectory was the intra-operatively modified trajectory based on EMG feedback.

Figure 3 illustrates a simple linear regression plot between the intra-operative EMG-based PTSE threshold vs. the post-operative programming PTSE threshold. The regression output shows that intra-operative EMG-based PTSE threshold was a statistically significant predictor variable for the post-operative programming PTSE threshold (*p* value <0.001). The regression model plot also demonstrates a linear relationship between the intra-operative CST and the post-operative PTSE threshold (R = 0.42). However, the R-squared value was 0.17, indicating the presence of trend between the two variables, although with significant variability or potential cofounders.

### Long-term follow up results

Post-operatively, no cases were found to have an abnormally low PTSE threshold which interfered with subsequent DBS programming. All patients completed a formal clinical assessment at the one year mark after the operation. The UPDRS part III score showed a mean improvement of 54% (SD 12.7%) which was similar to results of 52.7% (SD 12.7%) improvement by the pre-operative dopamine challenge test. The mean levodopa equivalent dose (LED) reduction was 59.5% (SD 23.5%). The mean stimulation parameter at one year were 2.84 mA and 2.70 mA respectively on right and left contacts (SD 0.6 and 0.7 mA), pulse width 60µS (SD 0) and frequency 115 Hz (SD 31 Hz). All results are summarized in Table 1.

### Representative case illustration

**Figure 4:**
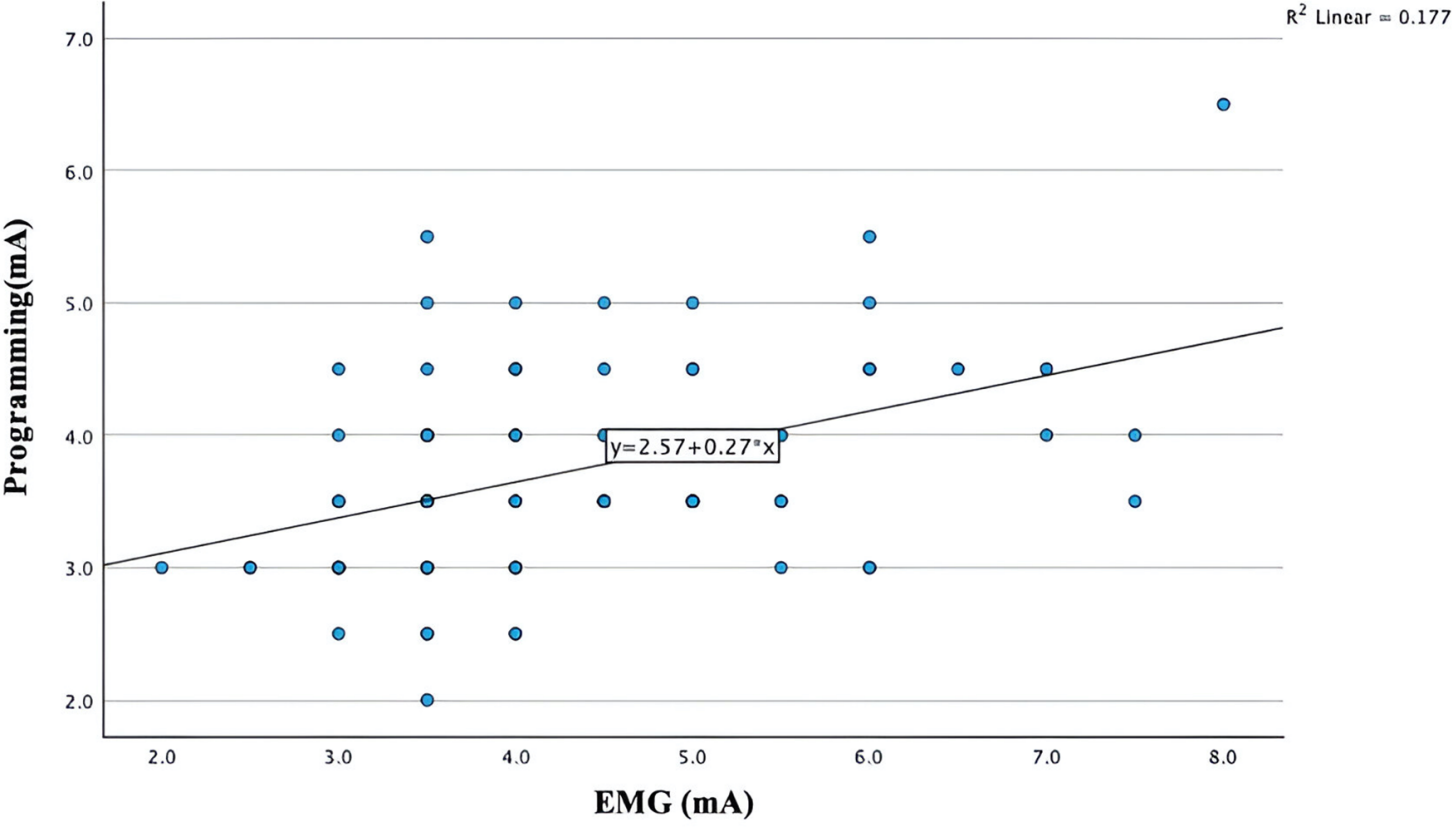
Scatter plot illustrating the EMG-based PTSE threshold vs programming PTSE threshold.

A case illustration was showed in Figure 4. Intra-operatively, macrostimulation of the original planned target induced contralateral limb EMG activation at 2.5 mA, which would be too low for an adequate therapeutic window for post-operative programming. Therefore, a 2nd track was explored 1 mm anterior and medially away from the 1st tract with its position remaining in the mediolateral centre of the STN. On repeated test stimulation, the EMG-based PTSE threshold was increased to 3.5 mA. During post-operative programming, the PTSE threshold was noted to be 3.5 mA with a clinical observation of slow gaze movement. The therapeutic window of stimulation was between 2.5–3 mA.

## Discussion

Our study demonstrated the feasibility of using intra-operative EMG under GA to predict the threshold of stimulation induced PTSE post-operatively. Firstly, we found that EMG from the contralateral limb was active in correlation to the increasing voltage of macrostimulation during STN DBS surgery under GA. Furthermore, in the majority of cases, the EMG-based PTSE threshold is slightly higher than the programming-based PTSE threshold. To the best of our knowledge, the present investigation is the first study using intra-operative EMG to guide intra-operative DBS lead placement under full GA.

Studies have been shown that the sweet spot of STN DBS corresponds to the sensory motor division of STN, which is located in the dorsolateral part of the nucleus (25). However, in our experience, a tract with typical STN signal together with limb movement synchronization on MER does not guarantee an adequate distance to the pyramidal tract which is immediately lateral to the STN. Moreover, there has been evidence stating that the pyramidal tract and STN can indeed be overlapped anatomically with each other (21). An overly lateral located DBS lead will induce pyramidal side effect at low stimulation intensity which will limit the effectiveness of DBS. When DBS is performed under LA, the side effect can be tested with clinical stimulation test. Under GA, the PTSE threshold can still be elicited though the activation of EMG but the sensitivity is different from that patient's objective feedback under LA. Our study showed that under GA, the EMG-based PTSE threshold from the contralateral upper limb was significantly higher than the post-operative programming PTSE threshold. The mean difference was 0.6 mA (SD: 1.1 mA, range: −2.0–4.0 mA).

Although a linear relationship (r = 0.42) was demonstrated between the predictor EMG threshold, and the dependent PTSE threshold, its association demonstrates limitations in strength (Figure 3). When comparing EMG-based PTSE thresholds to post-operative programming PTSE thresholds, we noted that over 76% of our results show that the EMG-based PTSE threshold is equal or higher than the programming threshold. Specifically, 90% of them are in a range of 0–2 mA, although few outliers with greater differences were also noted. This could potentially be explained through the individual variations of the impedance of focal brain tissue around the target during the operation and after the operation, as well as varying individual response to GA. From our experience, some patients may have continuous EMG activity of a low amplitude at baseline and BIS showing a deep hypnotic state without down-titrating targeted propofol concentration. In contrast, some patients may require the targeted propofol concentrations needed to be much reduced in order to increase the BIS between 40 and 60 and achieve adequate EMG signals.

In practice, when the intra-operative EMG-based threshold was too high or too low, i.e., <3 mA or >6 mA, a multifactorial approach is taken to reassessment the suitability of current lead implantation. Not only will we first review the targeting plan, we will also check the frame coordinates, review the MER signal and length, the depth of anaesthesia, and discuss any subsequent need for trajectory modifications with all members of the movement disorder team participating in the operation. If all other factors are deemed normal on assessment, our approach is to modify our trajectory away from the pyramidal tract for patients whom have a low EMG-based threshold. This approach is based on our center's experience, where a previous pilot study we performed showed that the intra-operative EMG-based threshold under GA-based DBS surgery was around 1 V higher than under LA, while the EMG-based threshold under LA was similar to the post-operative PTSE threshold. This was also demonstrated in Figure 4, where our planned trajectory was modified based on the intra-operative EMG-based threshold. Based on the result of the current study and our experience, we recommend that if the EMG-based threshold demonstrated under GA was lower than 3 mA, a new track away from the pyramidal tract should be considered so to avoid a low PTSE threshold in subsequent programming.

When evaluating the clinical outcomes of awake vs. asleep STN DBS, several studies have noted a high prevalence of adverse effects in those who performed asleep STN DBS without intra-operative neurophysiological monitoring i.e., balance disturbance, dysarthria. Given that there were no significant differences in stimulation intensity, some authors hypothesized that the PTSE threshold was impacted due to a potential suboptimal lead location in this group (8, 10). Studies have also found that approximately 20% of image-based targeting trajectories were found to be suboptimal, with intra-operative physiological monitoring improving lead localization (26). In our cohort, 7 of the 88 image-based trajectories (8%) had an initial low EMG-based threshold requiring new trajectories exploration during the operation. Our experience is consistent with the literature. The reason is likely be multifactorial, including limitations in image quality to clearly delineate boundaries of the pyramidal tract. There may also be error in the accuracy in application of the stereotactic frame, whereby literature quoted application accuracy could exceed beyond 1 mm (27). Furthermore, intra-operative brain shift may also account minor yet influential changes in lead accuracy. Therefore, we believe that for STN DBS under GA, intra-operative EMG can be of aid to help avoid lead placement error with resulting low post-operative PTSE.

## Limitations and future directions

Our study only uses the contralateral upper limb EMG as the indicator for PTSE. Yet, in clinical programming or in macrostimulation under LA, contralateral facial spasm, eye deviation and dysarthria are the more commonly reported PTSEs related to the location of the corticobulbar tract (2). Previously, Mahlknecht and colleagues used the orbicularis and dorsal interosseous muscle EMG with surface cup electrodes to detect the pyramidal tract activation in conjunction with motor evoked potentials and tractography in patients receiving chronic STN DBS (21). Their results showed that direct pyramidal tract activation can occur at stimulation thresholds that are within the range used in clinical routine. It also demonstrated that the corticobulbar tract was more anteriorly located than the pyramidal tract with diminishing distance caudally and a great level of overlap at the level of the STN. Theoretically, intra-operative facial EMG may be more representative of the programming PTSE. In our original pilot study with the patient awake, we adopted the use of both contralateral upper limb and facial EMG for monitoring. We noted that the upper limb EMG was a much more sensitive indicator for PTSE when compared to lower limb clinical and EMG response. Furthermore, despite its sensitivity, we noted that the facial EMG caused extreme discomfort due to need for insertion of needles to the patient's face, with subsequent risk of minor injuries such as bruising to the patient's face. Therefore, we decided to proceed without the facial EMG signal. Looking forward, we can study the use of facial EMG with surface cup electrodes to avoid the above named complications.

## Conclusion

During STN DBS lead implantation under GA, PTSE threshold can be tested by EMG via macrostimulation. It can provide real-time information on the laterality of the trajectory. This technique can be used as reference to guide the DBS lead placement and to prevent a low PTSE threshold in the postoperative chronic stimulation.

## Data Availability

The raw data supporting the conclusions of this article will be made available by the authors, without undue reservation.
